# Factors associated with "Ikigai" among members of a public temporary employment agency for seniors (Silver Human Resources Centre) in Japan; gender differences

**DOI:** 10.1186/1477-7525-4-12

**Published:** 2006-02-27

**Authors:** Kokoro Shirai, Hiroyasu Iso, Hideki Fukuda, Yasuhiro Toyoda, Toshio Takatorige, Kozo Tatara

**Affiliations:** 1 Graduate School of Social and Environmental Medicine, Osaka University, Japan; 2 Graduate School of Biomedical Science, Nagasaki University, Japan; 3 Department of Life and Welfare, University of the Air, Japan

## Abstract

**Background:**

"Ikigai" is culturally defined in the society of Japan as a comprehensive concept describing subjective well-being. It is considered to be related to life-satisfaction, self-esteem, morale, happiness as well as evaluation towards meaning of one's life. Although previous studies examined factors associated with Ikigai with smaller samples, consistent results have not been obtained, especially from the viewpoint of gender differences. Identification of gender-specific factors related with Ikigai among the elderly, may be of value to enhance subjective well-being.

**Methods:**

Self-administered questionnaires were distributed among 4,737 randomly selected members of the Silver Human Resources Centre (SHRC), a public temporary employment agency for seniors, in Osaka, Japan. This represents about 10% of all registered members (n = 41,593) in the 38 SHRC centres in Osaka. A total of 4,376 subjects (male: 2,913; female: 1,463) provided a satisfactory response to the questionnaire (response rate: 92%). The status whether they have "Ikigai" or not was evaluated by self-anchoring scale ranging from 0 to 5 (0 = lowest rate and 5 = highest rate of having "Ikigai"). Also, self-rated life-change score through work (-3 to 3) was evaluated by three items, i.e.) changes in (1) the number of friends through work, (2) social interests and (3) the quantity of conversation with others (1 = increase, 0 = no change, and -1 = decrease).

**Results:**

The factors associated with "Ikigai" for total subjects were the number of rooms in one's residence, annual income, healthy life style score (Breslow), the number of working days through SHRC, satisfaction with one's life history and life-change sore through work. The multivariable odds ratio (95%CI) of having "Ikigai" was 1.9 (1.1–3.3) for persons with no change in life thorough work compared with subjects with a score of ≦-1. Moreover, the multivariable odds ratios were 3.5 (1.9–6.6) for a life-change score = 1, 3.1 (1.7–5.7) for a score = 2 and 7.8 (4.0–15.2) for a score = 3 compared with persons with a score of ≦-1.

For male subjects, other factors associated with having "Ikigai" were the number of rooms in their residence, annual income, the number of working days through SHRC, subjective assessment of health condition, and degree of satisfaction with their life history. For female, the corresponding factors were the presence of a spouse and degree of satisfaction with their life history.

**Conclusion:**

Scores for life-changes through work were associated with a higher prevalence of having "Ikigai" for both male and female. For male, "Ikigai" tended to be associated with physical condition and socioeconomic factors such as the size of their residence or annual income, while for female, family relations such as having spouse and psychological factors such as satisfaction with one's life history were significant factors. In spite of the design limitations of this study, it is possible to conclude that the recognition of life change through obtaining work may enhance "Ikigai" among people who wish to engage in productive activities in their later stages of life for both male and female. SHRC has a potential to provide resources for fulfilling one's "Ikigai" through supporting working opportunities to realize life changes for both elder male and female.

## Background

Japan has experienced one of the largest increase in the proportion of persons aged 65 years and over in the world. The proportion of the elderly is 19.5% in 2005, and is expected to reach 25.2% in 2015 [1]. Health promotion and support for quality of life for the elderly is therefore an important task in such a society. In this connection, the idea that psychosocial conditions are related to the enhancement of health and reducing the risk of mortality in later stage of life is attracting growing attention. Most commonly, subjective well-being [2-5], social relations [6-10], participating productive activities and meaningful engagement towards one's life [11-14] have been studied as psychosocial factors associated with health and mortality of older populations.

These studies showed that lower levels of a subjective well-being and lack of a sense that life is worth living were associated with an increased risk of mortality. A subjective well-being and a feeling that life is worth living may represent prognostic factors for longevity and healthy aging in the later stages of life. A subjective well-being is often assessed with measures such as self-esteem, life satisfaction, self-concept, morale, depression, SF-36, QOL index, happiness and loneliness [15]. These measures represent evaluations of different aspects of well-being; for example, happiness and loneliness tend to constitute dimensions of emotional aspects, whereas the other assesses cognitive evaluation towards one's life [16].

However, "Ikigai" is understood as a comprehensive concept related to emotions, i.e) happiness and life-satisfaction, as well as to the cognitive evaluation of the meaning of one's life, self-esteem and self-efficacy.

In the present study, we focused on the concept of "Ikigai", which is culturally defined as a subjective evaluation of well-being among Japanese, and examined factors associated with "Ikigai", which is with an awareness of importance to use the term originated in the basis of cultural background and social norms [17]. Lyons (1998) refers to "Ikigai" as "What makes one feel good about oneself as a valued member of society who is in control of one's life", and Mathews (1996) stated that "Ikigai" is "What makes life worth living" [18]. More recently, some researchers have used the exact term "Ikigai" to report on its relationship with objective and subjective evaluation of health conditions [19-23]. It has also been reported that "Ikigai" varies among generations, and is modified by social activities and social networks [24-26].

The concept of "Ikigai" can be an important supportive element for health among the elderly [19-22], not only for those who are frail, but also for those who wish to maintain or promote their health and life. Traditional hypotheses of social gerontology, such as engagement theory and continuous theory supported the relationship with opportunities to engage in productive activity and subjective well-being in terms of concept of successful aging [3]. Therefore, we realize the importance of "Ikigai" among the elderly who are relatively fit and willing to participate in social activities, but are still struggling to find ways to enhance their quality of life.

We chose the subjects as elderly persons who were willing to engage in productive activities as members of the Silver Human Resources Centre (SHRC), which is one of the social resources for providing temporary work for the elderly. We hypothesized that to obtain work through SHRC may change their life and enhance "Ikigai" among the elderly. We therefore tried to explore the factors associated with "Ikigai" among those who are engaging in productive activities after their retirement age.

Also, previous studies indicated that gender differences in the evaluation of subjective well-being [26-28]. For example, male tended to report themselves as independent, achievement-oriented, financially-oriented, and more competitive than female [27,28], while female were more likely to describe themselves as sociable, moral, dependent and less assertive [28,29]. Furthermore, it was reported that female tended to have the greater access to sources of subjective well-being and to engage more in processes to protect the self than did male [30]. Those studies indicated that male and female show different presentation of their evaluation toward subjective well-being. Therefore, we specifically examined gender differences in factors associated with "Ikigai".

## Methods

### Study population

The survey was conducted among 4,737 randomly selected members of the Silver Human Resources Center (SHRC) in Osaka, Japan. There were a total of 41,593 members registered at the 38 SHRC centers in Osaka, from each of which about 10% of the registered members were randomly selected. A self-administered questionnaire was distributed after a full explanation of the purpose of the project had been given, and informed consent had been obtained from a total of 4,376 subjects (male, 2,913; female, 1,463) for a response rate of 92%. Relevant details about the subjects are shown in Table1 (see additional file 1).

The SHRC is a vocational introductory and placement agency for persons aged 60 years old and over in Japan since 1975. SHRC, supported in part by local and national governments, provides working opportunities for older individuals in order to support their fulfilment of life through engaging in productive activities. This institution provides job opportunities as paid work. However, the primary purpose of the institution is to enhance "Ikigai" though providing job opportunities as social activity not merely as economical activities.

### Measurement of psychosocial conditions

The concept of "Ikigai" was defined as "sense of life worth living" and the status whether they recognize themselves to have "Ikigai" or not was evaluated by self-anchoring scale [31] with a range from 0 to 5 (0 = lowest rate of having "Ikigai": meaning having no "Ikigai", and 5 = highest rate of having "Ikigai"). Usually, psychosocial factors are evaluated by means of scales consisting of multiple items to assess multidimensional aspects of certain feelings, conditions, cognitions or emotions. In our study, however, we assessed "Ikigai" by evaluating to what extent people themselves recognize that they have "Ikigai" or not. In addition, we employed a self-rated score for evaluating an awareness of changes in life in terms of relations with others through work (life-change score through work: -3 to 3) consisting of three items: (1) change in the number of friends as a result of working opportunity provided by SHRC; (2) change in social interests through working opportunity provided by SHRC and (3) change in the quantity of conversation with others through working opportunity provided by SHRC (increase = 1, no change = 0, decrease = -1).

Based on the result of the Cronbach α coefficient (α = 0.776), it was considered the validity of this score was acceptable. Other psychological conditions were investigated through the following questions: (1) Are you satisfied with what you have done trough your past life? ("Satisfaction with my life history"; yes = 1, no = 0); (2) Do you want to work in the community to use what you have learned and experienced to make a contribution to society? ("Wish to contribute to society"; yes = 1, no = 0); (3) Do you want to have time for yourself to relax in the later stages of your life? ("Wish to have time for myself"; yes = 1, no = 0)

### Related variables and statistical analysis

We used demographic, economic and health-related measures as covariates for the multivariable modelling process. For evaluation of health-related conditions, subjective assessment of health condition was scored from very bad (1) to very good (5). To dichotomize subjective assessment of health condition, "very good", "good" and "average" were categorized as good health. A healthy lifestyle was evaluated by using Breslow's 7 items (0–7). The number of days of hospitalization and of seeking medical consultation during the preceding year was self-reported. Due to the small number of subjects hospitalized (6.3% of the total), the hospitalization record was dichotomized into two categories (none = 0, more than 1 day = 1).

To assess financial circumstances, the number of rooms in the respondent's residence was used to evaluate housing property (1–2, 3, 4, 5, ≧6), and annual income including pension benefits was assessed in terms of Japanese yen (<1, 1–1.9, 2–3.9, 4–5.9, ≧6 million per year). In addition, satisfaction with one's living standard was evaluated in terms of the following categories: very satisfied, satisfied and not satisfied. The number of days (0–250) working trough SHRC during the preceding year was self-reported. The primary purpose of seeking work at SHRC was assessed as financial benefits (31.5%), health maintenance (39.5%), communicating with friends and business establishments (18.8%), learning new skills and knowledge (3.0%), contributing to society (5.9%) and other (1.3%). Because there were few responses for some of these categories, financial benefits, health maintenance and communicating with friends were considered to be the main purposes of seeking work and the rest were categorized as "other". Gender differences for the distribution and mean values of these factors were examined by means of Kruskal-Wallis and ANOVA test respectively.

For total, male and female subjects, linear regression analysis for "Ikigai" score (0 to 5) and multivariable-adjusted logistic regression analysis for having "Ikigai" ("Ikigai" score > 0) were used to determine factors associated with having "Ikigai". The presence of interaction with gender was tested by using cross-products in terms of gender and related factors. All analyses were conducted using SPSS^© ^ver.11.05J for Windows (SPSS Inc., Chicago, IL, USA). All P values for statistical tests were two-tailed and P < 0.05 was regarded as statistically significant.

## Results

Table 1 (see additional file 1) shows the characteristics of SHRC members enrolled in this study. Over 85.8% of the subjects were 60–74 years old. A higher proportion of female (26.9%) than male (3.8%) were living alone, while 34.7% of female and 60.1% of male were living with their spouse. As for financial status, 62.8% of male reported 2–3.9 million yen as annual income, whereas 43.7% of female reported less than 1 million yen as their annual income. However, differences in satisfaction with living standards between male and female were not significant, with 63.5% of male and 59.9% of female reporting to be satisfied. In terms of health, those who were hospitalized more than 1 day during the preceding year accounted for 7.1% of male and 4.8% of female, while the mean number of days for seeking medical consultation was 21 days for either gender. Male produced a higher healthy lifestyle score (mean value = 3.6) than female (mean value = 3.5). The mean number of days of work provided by SHRC was higher for male (mean value = 99.9 days) than for female (mean value = 86.6 days). The proportion of a score of 3 for changes in life style as a result of work (all 3 categories increased positively) was 31.3% for male and 33.4% for female.

Table 2 (see additional file 2) shows factors associated with "Ikigai" score (0–5) as determined by linear regression analysis. Factors associated with "Ikigai" were age, sex, having a spouse, the number of rooms, annual income, healthy lifestyle, the number of working days through SHRC, purpose of work, satisfaction with one's life history, a wish to have time for oneself and self-rated life-changes score through work (adjusted R^2 ^= 0.20; p < 0.001). Among male, the significant factors were the number of rooms in one's residence, annual income, healthy lifestyle, purpose of work (for financial benefit), purpose of work (for financial maintenance), satisfaction with one's life history, wish to contribute to society, wish to have time for oneself and score for life-changes through work. For female the factors associated with "Ikigai" were age, number of rooms in one's residence, healthy lifestyle, satisfaction with one's life history and score for life-changes through work. Subjective assessment of health condition was not associated with "Ikigai" for either sex.

**Figure 1:**
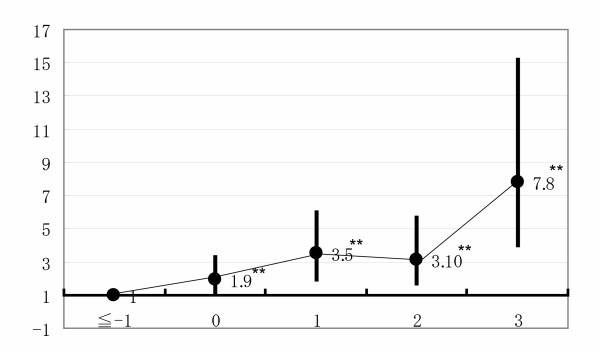
**Multivariable odds ratios of having "Ikigai" according to life-change scores through work**. **P for trend < 0.001

Factors related to having "Ikigai" (no = 0; yes ≧1) were examined further by means of logistic regression analysis (Table 3 [see additional file 3]). Overall, persons with a higher score for life-change through work showed a higher odds ratio for having "Ikigai" (Figure 1). In comparisons with persons with a score of ≦-1, the multivariable odds ratio (95%CI) for having "Ikigai" was 1.9 (1.1–3.3) for a score of 0, 3.5 (1.90–6.6) for a score of 1, 3.1 (1.70–5.7) for a score of 2, and 7.8 (4.02–15.2) for a score of 3. Satisfaction with one's life history and wish to have time for oneself were also related to having "Ikigai". Other associated factors were a better subjective assessment of health, a higher healthy lifestyle score, a larger number of rooms in one's residence, a higher annual income and a greater number of working days through SHRC.

For male, the factors associated with having "Ikigai" were a larger number of rooms, a higher annual income, a greater number of working days through SHRC, better subjective health condition a higher healthy lifestyle score, satisfaction with one's life history and a higher self-rated life-change score. For female, the corresponding factors were the presence of a spouse, satisfaction with one's life history and a higher self-rated life-change score. There was a significant gender difference in the association between annual income and "Ikigai" (P for interaction <0.05).

## Discussion

The score for life-changes through work was associated with a higher prevalence of having "Ikigai" for both male and female. Furthermore, our study suggested there were several gender differences for factors associated with "Ikigai". For male, physical condition and socioeconomic factors such as annual income, the number of rooms in one's residence and work for financial benefit were closely associated with "Ikigai", while for female family relations such as having a spouse and psychological factors were associated factors.

A meta-analysis by Pinquart and Sorensen (2000) [30] reported supportive results of our study. It mentioned that social and environmental situations were more strongly related to life satisfaction and happiness among male, while social integration was more strongly related among female. Piquart & Sorensen (2001) [15] also reported that income was associated with happiness for male but not for female. Furthermore, our results are consistent with those for community residents aged 60 and over as reported by Fujimoto et al. (2004) [26]. They also suggested that male showed a closer relationship with physical condition, having an occupation and playing a role in society, while female showed a closer connection between "Ikigai" and family and psychological factors, such as less depressive symptoms (GDS) and more life satisfaction (LSI-K). Moreover, although the gender differences observed in our study reached statistical significance only for annual income, we examined them after controlling potential confounding factors, while previous studies examined gender differences only descriptively and did not examined interactions.

Male but not female showed a significant association between working days and "Ikigai". Furthermore, both male and female showed significant relations of life change score, as well as satisfaction on one's life history with "Ikigai". This suggests that not only obtaining work, but also being a member of SHRC may increase opportunities to meet people, to have conversations with others and to contact with social events, leading to obtain "Ikigai". In this study, it was not possible to explore structural pass to understand how they recognize "Ikigai". However, it may be possible to imply that SHRC provides a positive influence towards the elderly who wish to engage in work in their late stage of life. In other word, SHRC will be considered as one of the rewardable social resources in the aged society. Furthermore, since male and female showed different factors associated with "Ikigai", SHRC should provide the elderly a variety of types and periods of work, such as paid work, non-paid work as well as volunteer work according to their wish.

## Conclusion

Since this study was designed as a cross-sectional study, it was not possible to determine the causal effect of working opportunities obtained through SHRC on having "Ikigai". Furthermore, this study did not have a control group of not having work opportunities at all in the same generation. However, within the members of people who have chance to engage in productive activities, we found a significant dose-response relationship between the number of working days through SHRC and "Ikigai" among both male and overall subjects. Moreover, both male and female reported having more "Ikigai" when they became aware of positive changes in their life in terms of relations with others through participating in productive activities, such as having more frequent conversation with people, gaining greater interest in life, and getting more friends through work opportunities. Therefore, in spite of the limitations of the study design, it can be concluded that recognition of life change through obtaining work may enhance "Ikigai" among people who wish to engage in productive activities in the later stages of life. A longitudinal study or a clinical trial will be necessary to confirm this finding. Our findings also suggest that both male and female can increase their "Ikigai" through different pathways which warrant further research. SHRC has demonstrated its potential to provide resources for enhancing "Ikigai" through providing work opportunities for both elderly male and female.

## Authors' contributions

Kokoro Shirai participated in the study concept and design, acquisition of data, analysis and interpretation of data, and drafting of the manuscript. Hiroyasu Iso participated in analysis and interpretation of data, and the help for drafting of the manuscript, and provided statistical expertise. Hideki Fukuda participated in the study concept and design, acquisition of data, analysis of data and interpretation of data, and the help for drafting of the manuscript. Yasuhiro Toyoda helped for critical revision of the manuscript. Toshio Takatorige participated in the study concept and design, acquisition of data and the help for critical revision of the manuscript. Kozo Tatara participated in the study concept and design, acquisition of data, interpretation of data, and the help for critical revision of the manuscript and supervised the conduct of the study.

## Supplementary Material

Additional File 1 Table 1: Characteristics of the members of Silver Human Resource Center (SHRC) in Japan Click here for file

Additional File 2 Table 2: Factors associated with "Ikigai" score Click here for file

Additional File 3 Table 3: Odds ratio (95% CI) of having "Ikigai" by gender Click here for file
