# Supplementary material for: Factors associated with "Ikigai" among members of a public temporary employment agency for seniors (Silver Human Resources Centre) in Japan; gender differences
Source: Health Qual Life Outcomes. 2006 Feb 27;4:12. doi: 10.1186/1477-7525-4-12 (PMC1450260; doi:10.1186/1477-7525-4-12)
Supplement: Additional File 1 — Table 1: Characteristics of the members of Silver Human Resource Center (SHRC) in Japan [file 1477-7525-4-12-S1.pdf]

Table1 Characteristics of the members of Silver Human Resouces Center (SHRC) in Japan N %

		Total		Male		Female		gender difference
Age	< 60	73	1.7	20	0.7	53	3.6	***
	60-64	1161	26.6	726	25.0	435	29.8	
	65-69	1545	35.3	1058	36.4	487	33.3	
	70-74	1043	23.9	716	24.6	327	22.4	
	75-79	424	9.7	305	10.5	119	8.1	
	80-89	123	2.8	83	2.9	40	2.7	
	≧90	2	0.0	1	0.0	1	0.1	
Family	living alone	504	11.6	113	3.9	391	26.9	***
	with spouse	2241	51.6	1737	60.1	504	34.7	
	2 generations	796	18.3	456	15.8	340	23.4	
	3generations	81	1.9	64	2.2	17	1.2	
	others	719	16.6	518	17.9	201	13.8	
Number of rooms in one's residence (rooms)	1-2	482	11.0	250	8.6	232	15.9	***
	3	1114	25.5	699	24.1	415	28.4	
	4	993	22.8	694	23.9	299	20.5	
	5	1011	23.2	705	24.3	306	20.9	
	≧6	764	17.5	555	19.1	209	14.3	
Annual income including pension benefits (million yen)	< 1	841	19.7	228	7.9	613	43.7	***
	1-1.9	1121	26.2	543	18.9	578	41.2	
	2-3.9	1989	46.5	1803	62.8	186	13.3	
	4-5.9	291	6.8	270	9.4	21	1.5	
	≧6	31	0.7	27	0.9	4	0.3	
Satsfaction with one's living standard	very satisfy	721	16.8	477	16.7	244	17.1	n.s
	satisfy	2670	62.3	1814	63.5	856	59.9	
	not satisfy	895	20.9	567	19.8	328	23.0	
Subjective assessment of health condition	very bad	24	0.6	17	0.6	7	0.5	***
	bad	312	7.2	212	7.3	100	6.9	
	average	2241	51.6	1522	52.7	719	49.6	
	good	912	21.0	615	21.3	297	20.5	
	very good	850	19.6	522	18.1	328	22.6	
Hospitalization during the past year	none	4100	93.7	2705	92.9	1395	95.4	***
	≧1	276	6.3	208	7.1	68	4.6	
Seeking medical consultation during the		Mean±SD=21.63±35.36		Mean±SD= 21.63±35.3		Mean±SD=21.64±35.49		n.s
Healthy lifestyle score (Breslow: 0-7)		Mean±SD=3.58±1.89		Mean±SD=3.62±1.9		Mean±SD=3.50±1.86		**
Numbers of working days through SHRC (0-250 days)		Mean±SD=95.58±75.21		Mean±SD=99.86±76.02		Mean±SD=86.63±72.74		***
Initial purpose of work through SHRC	financial benefit	1332	31.5	887	31.4	445	31.6	n.s
	health maintenance	1674	39.5	1166	41.2	508	36.1	
	communicating with friends	795	18.8	475	16.8	320	22.7	
	others	434	10.2	300	10.6	134	9.5	
Life-change score through work (-3,-2,-1, 0, 1, 2, 3)	-3	30	0.7	20	0.7	10	0.7	**
	-2	44	1.1	38	1.4	6	0.4	
	-1	128	3.1	98	3.5	30	2.2	
	0	1055	25.5	715	25.6	340	25.3	
	1	762	18.4	512	18.4	250	18.6	
	2	792	19.2	533	19.1	259	19.3	
	3	1322	32.0	873	31.3	449	33.4	
Satisfaction with my life history	no	674	16.2	475	17.0	199	14.5	**
	yes	3495	83.8	2319	83.0	1176	85.5	
Wish to contribute to society	no	3151	72.0	2070	71.1	1081	73.9	**
	yes	1225	28.0	843	28.9	382	26.1	
Wish to have time for myself	no	3433	78.5	2261	77.6	1172	80.1	*
	yes	943	21.5	652	22.4	291	19.9	
Total		4376	100	2913	100	1463	100	

Gender difference were examined by kruskal wallis and ANOVA

*p < 0.1 ** p < 0.05 ***p < 0.001
