# Supplementary material for: Factors associated with "Ikigai" among members of a public temporary employment agency for seniors (Silver Human Resources Centre) in Japan; gender differences
Source: Health Qual Life Outcomes. 2006 Feb 27;4:12. doi: 10.1186/1477-7525-4-12 (PMC1450260; doi:10.1186/1477-7525-4-12)
Supplement: Additional File 2 — Table 2: Factors associated with "Ikigai" score [file 1477-7525-4-12-S2.pdf]

Table2 Factors associated with "Ikigai" score

variables	Total			Male			Female		
	SE	β		SE	β		SE	β	
Age (year)	0.02	-0.04	***	0.03	-0.01		0.04	-0.08	***
Sex (male=1, female=0)	0.07	-0.20	***						
Spouse (yes=1)	0.07	0.04	**	0.10	0.03		0.09	0.02	
Number of rooms in one's residence (rooms)	0.02	0.07	***	0.02	0.06	***	0.03	0.07	**
Annual income including pension benefits (mill	0.03	0.09	***	0.04	0.11	***	0.06	0.03	
Subjective assessment of health condition (1-	0.03	0.02		0.03	0.02		0.05	0.02	
Hospitalization during the past year (yes=1)	0.10	0.01		0.11	0.01		0.21	0.00	
Healthy lifestyle score (Breslow: 0-7)	0.01	0.26	***	0.02	0.24	***	0.02	0.29	***
Numbers of working days through SHRC (０-250	0.02	0.02		0.03	0.03		0.04	0.01	
Purpose of work (for financial benefit=1)	0.07	-0.08	***	0.08	-0.11	***	0.11	-0.04	
Purpose of work (for health=1)	0.06	-0.05	***	0.07	-0.07	***	0.11	-0.01	
Life-change score through work (-3 to 3)	0.02	0.17	***	0.02	0.18	***	0.03	0.16	***
Satisfaction on my life history (yes=1)	0.08	0.10	***	0.09	0.09	***	0.14	0.11	***
Wish to contribute to society (yes＝１)	0.06	0.03		0.08	0.04	*	0.11	-0.01	
Wish to have time for myself (yes＝１)	0.07	-0.05	***	0.08	-0.04	*	0.12	-0.05	*
Adjusted R^2		0.20			0.19			0.17	

* p < 0.1 ** p < 0.05 ***p < 0.001
