# Supplementary material for: Factors associated with "Ikigai" among members of a public temporary employment agency for seniors (Silver Human Resources Centre) in Japan; gender differences
Source: Health Qual Life Outcomes. 2006 Feb 27;4:12. doi: 10.1186/1477-7525-4-12 (PMC1450260; doi:10.1186/1477-7525-4-12)
Supplement: Additional File 3 — Table 3: Odds ratio (95% CI) of having "Ikigai" by gender [file 1477-7525-4-12-S3.pdf]

Table3:Odds ratio (95% CI) of having "Ikigai" by gender

Variables		Total n	Total Odds ratio	Total 95% CI	Male n	Male Odds ratio	Male 95% CI	Female n	Female Odds ratio	Female 95% CI
Age	<65	1146	1.0		682	1.0		464	1.0	
	65-69	1444	1.0	(0.68- 1.6)	988	1.2	(0.76- 2.0)	456	0.7	(0.27- 1.7)
	≧70	1461	0.8	(0.53- 1.2)	1014	0.9	(0.58- 1.5)	447	0.5	(0.22- 1.3)
Spouse (yes=1)		894	1.0		276	1.0		618	1.0	
		3132	1.3	(0.86- 2.0)	2392	1.0	(0.52- 1.8)	740	1.9	(0.90- 3.9) *
Number of rooms in one's residence (rooms)	1-2	420	1.0		214	1.0		206	1.0	
	3	1022	2.0	(1.19- 3.5)**	635	2.0	(1.07- 3.9) *	387	2.1	(0.78- 5.8)
	4	923	1.5	(0.87- 2.5)	641	1.5	(0.82- 2.9)	282	1.3	(0.45- 3.6)
	5	947	1.6	(0.93- 2.7) *	655	1.7	(0.91- 3.4) *	292	1.3	(0.48- 3.4)
	≧6	731	1.9	(1.02- 3.6) *	532	2.2	(1.06- 4.7) *	199	1.1	(0.34- 3.7)
Annual income including pension benefits (million yen)	<1 mill	756	1.0		188	1.0		568	1.0	
	1-3.9 mill	2897	1.5	(0.93- 2.3) *	2169	1.62	(0.88- 2.98)	728	1.5	(0.76- 3.1)
	≧4 mill	313	2.3	(0.93- 5.6) *	290	2.78	(0.99- 7.8) *	23	0.8	(0.08- 7.6)
Subjective assessment of health condition	verbad &bad	297	1.0		202	1.0		95	1.0	
	very good, good & average	3748	0.9	(0.54- 1.57)	2479	1.2	(0.96- 1.5) *	1269	1.2	(0.81- 1.7)
Hospitalization during the past year	none	3805	1.0		2497	1.0		1308	1.0	
	≧1	250	1.2	(0.63- 2.4)	190	1.4	(0.65- 3.1)	60	0.5	(0.14- 2.1)
Healthy lifestyle score (Breslow: 0-7)	0	48	1.0		26	1.0		22	1.0	
	1-3	1924	2.8	(0.77- 10.3)	1233	3.8	(0.73- 20.1)	691	2.1	(0.22- 20.2)
	4-5	1323	3.1	(0.83- 11.2) *	914	3.8	(0.73- 20.2)	409	2.8	(0.28- 28.0)
	6-7	760	4.2	(1.07- 16.0) *	514	4.5	(0.81- 24.7) *	246	6.7	(0.56- 80.4)
Numbers of working days through SHRC (days)	none	251	1.0		171	1.0		80	1.0	
	1-99	1407	1.3	(0.78- 2.3)	873	1.7	(0.89- 3.1)	534	0.7	(0.18- 2.5)
	≧100	1554	1.7	(0.96- 3.0) *	1113	1.8	(0.98- 3.5) *	441	1.2	(0.30- 4.9)
Purpose of work (for financial benefit=1)	no	2849	1.0		1894	1.0		955	1.0	
	yes	1206	0.9	(0.60- 1.4)	793	1.0	(0.59- 1.6)	413	0.9	(0.35- 2.2)
Purpose of work (for health=1)	no	2477	1.0		1590	1.0		887	1.0	
	yes	1578	1.4	(0.91- 2.2) *	1097	1.5	(0.91- 2.6) *	481	1.2	(0.48- 2.8)
Life-change score through work	≦-1	169	1.0		130	1.0		39	1.0	
	0	947	1.9	(1.12- 3.3)**	637	2.2	(1.21- 4.0)**	310	1.2	(0.30- 4.8)
	1	713	3.5	(1.90- 6.6)**	476	3.4	(1.72- 6.8)**	237	3.6	(0.75- 17.6)
	2	741	3.1	(1.70- 5.7)**	493	2.6	(1.34- 4.9)**	248	6.9	(1.20- 39.3)**
	3	1289	7.8	(4.02- 15.2)**	853	13.1	(5.60- 30.5)**	436	2.8	(0.64- 12.0)
Satisfaction with my life history	no	574	1.0		400	1.0		174	1.0	
	yes	3336	3.1	(1.94- 5.0)**	2206	3.7	(2.04- 6.6)**	1130	2.3	(0.98- 5.5) *
Wish to contribute to society	no	2880	1.0		1881	1.0		999	1.0	
	yes	1175	0.8	(0.50- 1.4)	806	0.7	(0.35- 1.2)	369	1.3	(0.50- 3.2)
Wish to have time for myself	no	3176	1.0		2083	1.0		1093	1.0	
	yes	879	0.6	(0.39- 1.0)**	604	0.5	(0.27- 0.9)	275	1.4	(0.51- 4.0)
Total		4376			2913			1463		

* p < 0.1 **p < 0.05
